# Compliance to the recommended use of folic acid supplements for women in Sweden is higher among those under treatment for infertility than among fertile controls and is also related to socioeconomic status and lifestyle

**DOI:** 10.1080/16546628.2017.1334483

**Published:** 2017-06-09

**Authors:** Tiina Murto, Agneta Yngve, Agneta Skoog Svanberg, Signe Altmäe, Andres Salumets, Kjell Wånggren, Anneli Stavreus-Evers

**Affiliations:** ^a^ Department of Women’s and Children’s Health, Uppsala University, Uppsala, Sweden; ^b^ Department of food, nutrition and dietetics, Uppsala University, Uppsala, Sweden; ^c^ Competence Centre on Health Technologies, Tiigi 61b, Tartu, Estonia; ^d^ Institute of Clinical Medicine, Department of Obstetrics and Gynecology, University of Tartu, Tartu, Estonia; ^e^ Institute of Bio- and Translational Medicine, University of Tartu, Tartu, Estonia; ^f^ Department of Obstetrics and Gynecology, University of Helsinki and Helsinki University Hospital, Helsinki, Finland; ^g^ Department of Clinical Science, Intervention and Technology, Karolinska Institutet, Huddinge, Sweden

**Keywords:** Folic acid, folate, infertility, socioeconomic status, lifestyle

## Abstract

**Background**: Folate has been discussed in relation to fertility among women, but studies on women under treatment for infertility are lacking.

**Objective**: The objective of this study was to investigate folic acid supplement use and folate status among women under treatment for infertility (hereafter infertile) and fertile women also in regard to socioeconomic and lifestyle factors.

**Design**: Lifestyle and dietary habits, and use of dietary supplements were assessed using a questionnaire. Blood samples were obtained for analysis of folate status. 24-hour recall interviews were also performed.

**Results**: Highly educated, employed and infertile women were most prone to using folic acid supplements. The infertile women had a significantly better folate status than the fertile women. Folate status did not correlate with socioeconomic or lifestyle factors. The infertile women were physically more active, smoked less and were employed. Our questionnaire data had only fair agreement with the data from 24-hour recalls, but the folate status data was clearly correlated to our questionnaire results.

**Conclusions**: Infertile women were most prone to using folic acid supplements and had better folate status than the controls. High educational and employment status were found to be key factors for high compliance to the recommended use folic acid supplements.

## Introduction

It is commonly known that preconceptional folic acid supplementation prevents neural tube and other birth defects [1,2]. Therefore, public health authorities all over Europe, including Sweden, recommend an intake of 400 µg folate per day for women of reproductive age [1,3]. Despite several public health campaigns and recommendations for over two decades, the compliance to folic acid supplementation is low in many countries. Results of previous studies have shown that less than 50% of women planning a pregnancy use preconceptional folic acid supplementation [4–7]. The current Swedish recommendation regarding supplement use for those planning a pregnancy is to take a supplement of 400 µg folic acid per day, while pregnant or lactating women should aim for a total of 500 µg per day of folate or folic acid, combining supplements and folate rich foods [3].

Previous studies of infertile women have shown that preconceptional folic acid supplementation increases folate levels and decreases levels of the amino acid homocysteine (Hcy) in follicular fluid [8]. In addition, folic acid supplementation has been related to better embryo quality and chance of pregnancy [9], and one study of women taking multivitamins including folic acid showed a reduced risk of ovulatory infertility [10]. It is known that infertile women use more folic acid supplements than fertile women, but there is evidence that even though over 80% of infertile women are aware of the benefits of using folic acid supplements, only half actually use them [11,12].

Age, socioeconomic status and educational level have been related to the awareness and use of folic acid supplements [13–15]. However, to our knowledge, such studies related to infertile women are lacking. Furthermore, for studies on diet and dietary supplement intake, the method of data collection is of importance. Most studies have involved the use of questionnaires for assessment of vitamin intake from diet alone and information on the intake of dietary supplements is lacking. Additionally, studies involving questionnaires for assessing actual intake of supplements have rarely been validated by using alternative methods such as 24-hour recall interviews [16] and rarely combined with folate status assessment.

The aim of this study was to investigate folic acid supplement use and folate status in infertile and fertile women also in regard to socioeconomic and lifestyle factors. In addition, a sub-analysis of 24-hour recall data compared with questionnaire data was performed to validate the used questionnaire.

## Materials and methods

### Study subjects and design

In this prospective observational study, women referred for infertility treatment were recruited. The women attended the Fertility Unit, Karolinska University Hospital, Huddinge, Sweden, between 2005 and 2007, or the Centre for Reproduction at Uppsala University Hospital, Sweden between 2008 and 2010. Healthy, proven fertile, non-pregnant and non-lactating women from the same geographic area were randomly recruited as controls.

There were 360 infertile women who agreed to participate. Of these women, 340 (94.4%) women completed the questionnaire. Of the 199 proven fertile women included in the study, 188 (94.4%) women completed the questionnaire. In total, 528 women were included in the final analyses, of which 340 were women with various infertility diagnoses (Table 1), and 188 were healthy, fertile women in the control group.

**Table 1.** Data of infertility diagnoses. Study population n = 340.

Diagnosis	n (%)
Unexplained infertility	180 (52.9)
Male infertility	86 (25.3)
Female infertility	74 (21.8)

### Questionnaire

A questionnaire was used to assess socioeconomic status, health, lifestyle and dietary habits as well as the use of dietary supplements. The questions included age, place of birth, marital status, education and employment status. Self-reported height and weight were converted to BMI (kg/m^2^), and categorized as underweight (< 18.5), normal weight (18.5–24.9), overweight (25.0–29.9) and obese (≥ 30). There were also questions about physical activity and the participants’ perceived subjective health, as well as smoking habits and alcohol use.

There were three options as regards the question on use of dietary supplements: yes, sometimes and no. A list, containing the 22 most commonly used dietary supplements in Sweden, was included. An open-ended question, where the participants could specify what kind of supplement(s) they used if not on the list, was included. If taking a folic acid supplement, the participants answering ‘yes’ or ‘sometimes’ were recorded as being folic acid supplement users and they could self-report the amount (µg) of folic acid ingested. The supplement content of folic acid was approximated from product information. The mean value of folic acid intake was calculated if several folic acid-containing supplements were used alternatively.

### 24-hour recall

For assessment of dietary folate intake, 24-hour recall interviews were performed, and these included 103 of the study subjects. The interviews were performed twice and only data from women participating in both interviews were included for further analyses (n = 91). The subjects were asked to recall in as much detail as possible what they had been eating and drinking from the time they woke up the day before, to 24 hours later. To help estimate portion sizes, the Swedish Food Administration food model ‘Matmallen’ was used. All information that could possibly facilitate the registration of data was collected, i.e. brands, fat content and preparation method. The subjects were also asked to recall supplement intake. Dietary information from the 24-hour recall interviews was converted to grams through use of a conversion table. When brands were indicated, internet sites and/or telephone numbers for product information served to assist in obtaining information about food composition as precisely as possible. The reported food, beverage and dietary supplement intakes were entered in the dietary analysis program Stor MATs 406 (Rudans Lättdata, Västerås, Sweden), which uses the Swedish food composition database (version 02_1; National Food Administration, Uppsala, Sweden).

To reveal possible under- and over-reporting in 24-hour recall interviews, the Goldberg cut-off equation was used. Only normal-reported interviews were included in the study. There were three cases of missing data, seven cases of under-reporting and six cases of over-reporting, resulting in 75 cases for the final analyses.

### Validation of the questionnaire

In order to validate the questionnaire in regards to folic acid supplement use, the results from two 24-hour recall interviews on actual supplement use were compared with the data from questionnaires providing usual supplement use in 75 cases.

### Folate and homocysteine analysis

Blood samples were obtained for assay of plasma folate and Hcy. After centrifugation, plasma was stored at −70 °C until analysis. Two-step luminescence competitive analysis involving folate-binding protein and acridinium ester-labelled folate was used for assay of folate. Homocysteine was reduced to nicotinamide adenine dinucleotide, which was measured at 450 nm. The result was proportional to the concentration of Hcy. Haemolytic plasma samples or folate values > 90 nmol/L were excluded because of the risk of misleading values. The reference values for plasma folate were >8 nmol/L(Architect, ABBOTT) and for plasma homocysteine < 15 µmol/L [17].

### Statistical analysis

Statistical analyses were performed using IBM SPSS Statistics 20.0 software (IBM Corporation, NY, USA). For comparisons involving categorical variables, the χ^2^ test or Fisher’s exact test (for less than five samples) was applied. Pearson’s analysis was used to investigate correlation. Cohen’s kappa coefficient was used to assess differences in folic acid supplement use when handling data from the questionnaires and data from the 24-hour recall interviews. Principal component analysis (PCA) was performed using SIMCA software (Umetrics, Umeå, Sweden). PCA was used for analyses of socioeconomic and lifestyle factors.

## Results

### Folic acid supplement use and folate status in relation to socioeconomic and lifestyle factors

Among infertile women, folic acid supplement users had a significantly higher educational level than non-users (p = 0.037) (Table 2). When comparing folic acid supplement users and non-users in the total population of women, the folic acid supplement users had higher educational level (p = 0.017) as well as employment status (p = 0.026) (Table 2). Not surprisingly, significantly more infertile women used folic acid supplements (73.2% vs. 39.4%, p < 0.0001), and had significantly higher mean daily folic acid supplement intake (p < 0.0001) compared with the fertile women (Table 3). Higher mean dietary folate intake was also related to folic acid supplement use in infertile women (p = 0.020) (Table 3). Folic acid supplement use was evidently related to a better folate status (Table 3). However, Pearson’s correlation analysis did not show correlations between socioeconomic and lifestyle factors vs. folate status.

**Table 2.** Socioeconomic and lifestyle factors in infertile and fertile folic acid (FA) supplement users and non-users. Data are given as n(%).

	Infertile n = 33			Fertile n = 187			All n = 525		
	FA supplement n = 249	No FA supplement n = 89	p-value	FA supplement n = 74	No FA supplement n = 113	p-value	FA supplement n = 323	No FA supplement n = 202	p-value
Age (years)			0.456			0.876			0.344
22–34	139 (55.8)	54 (60.7)		25 (33.8)	40 (35.4)		164 (50.8)	94 (46.5)	
35–44	110 (44.2)	35 (39.3)		49 (66.2)	73 (64.6)		159 (49.2)	108 (53.5)	
BMI			0.126			0.711			0.197
Underweight (< 18.5)	4 (1.6)	2 (2.2)		1 (1.3)	5 (4.4)		5 (1.5)	7 (3.5)	
Normal weight (18.5–24.9)	183 (73.5)	53 (59.5)		54 (73.0)	79 (69.9)		237 (73.4)	132 (65.3)	
Overweight (25.0–29.9)	44 (17.7)	24 (27.0)		14 (18.9)	23 (12.3)		59 (18.3)	47 (23.3)	
Obese (≥ 30)	17 (6.8)	9 (10.1)		2 (2.7)	3 (2.6)		19 (5.9)	12 (5.9)	
Origin			0.186			0.664			0.415
Swedish	223 (89.5)	75 (84.3)		63 (85.1)	99 (87.6)		286 (88.5)	174 (86.1)	
Other	26 (10.4)	14 (15.7)		11 (14.9)	14 (12.4)		37 (11.5)	28 (13.9)	
Marital status			0.239			0.110			0.001
Married	118 (47.4)	43 (48.3)		38 (51.3)	63 (55.7)		156 (48.3)	106 (52.5)	
Living together	131 (52.6)	45 (50.1)		29 (39.2)	32 (28.3)		160 (49.5)	77 (38.1)	
Separated	0 (0)	0 (0)		2 (2.7)	10 (8.8)		2 (0.6)	10 (5.0)	
Registered partnership	0 (0)	1 (1.1)		0 (0)	0 (0)		0 (0)	1 (0.5)	
Education			0.037			0.356			0.017
≤ 12 years	77 (30.9)	39 (43.8)		24 (32.4)	45 (39.8)		101 (31.3)	84 (41.6)	
> 12 years	172 (69.1)	50 (56.2)		49 (66.2)	68 (60.2)		221 (68.49	118 (58.4)	
Employment status			0.323			0.347			0.026
Working	230 (92.4)	78 (87.6)		54 (73.0)	88 (77.9)		284 (87.9)	166 (82.2)	
Not-working	13 (5.2)	5 (5.6)		11 (14.9)	10 (8.8)		24 (7.4)	15 (7.4)	
Studying	6 (2.4)	5 (5.6)		7 (9.5)	15 (13.0)		13 (4.0)	20 (9.9)	
Physical activity			0.512			0.692			0.223
Never	6 (2.4)	4 (4.5)		7 (9.5)	9 (8.0)		13 (4.0)	13 (6.4)	
< Once/week	83 (33.3)	26 (29.2)		22 (29.7)	28 (24.8)		105 (32.5)	54 (26.7)	
≥ Once/week	158 (63.4)	58 (65.2)		45 (60.8)	75 (66.4)		203 (62.8)	133 (65.8)	
Subjective health			0.201			0.164			0.859
Excellent	29 (11.6)	13 (14.6)		7 (9.4)	14 (12.4)		36 (11.1)	27 (13.4)	
Good	198 (79.5)	63 (70.8)		58 (78.4)	91 (80.5)		256 (79.3)	154 (76.2)	
Fair	19 (7.6)	10 (11.2)		5 (6.8)	6 (5.3)		24 (7.4)	16 (7.9)	
Poor	1 (0.4)	2 (2.2)		3 (4.0)	0 (0)		4 (1.2)	2 (1.0)	
Smoking			0.852			0.844			0.913
Yes	21 (8.4)	7 (7.9)		11 (14.9)	18 (15.9)		289 (89.5)	177 (87.6)	
No	226 (90.8)	82 (92.1)		63 (85.1)	95 (84.1)		32 (9.9)	25 (12.4)	
Alcohol (glasses/week)			0.414			0.982			0.390
≥ 9	17 (6.8)	4 (4.5)		7 (9.5)	11 (9.7)		24 (7.4)	15 (7.4)	
< 9	214 (85.9)	80 (89.9)		65 (87.8)	101 (89.4)		279 (86.4)	81 (89.6)

**Table 3.** Folic acid (FA) supplement daily intake (according to questionnaire) and daily dietary folate intake (assessed by 24-hour recalls), and folate status according to plasma folate and plasma homocysteine (Hcy) in infertile and fertile women. Data are shown as means ± SD.

	Infertile			Fertile			Infertile	Fertile	
	FA supplement n = 249	No FA supplement n = 89	p-value	FA supplement n = 74	No FA supplement n = 113	p-value	n = 338	n = 187	p-value
FA supplement intake µg/day	413.97 ± 142.72	-		401.02 ± 136.89	-		298.53 ± 221.91	156.07 ± 213.70	< 0.0001
Dietary folate intake µg/day	164.93 ± 89.57	139.98 ± 65.63	0.020	150.56 ± 78.35	160.51 ± 74.01	0.382	158.26 ± 84.28	156.70 ± 75.49	0.835
Plasma folate nmol/L*	26.9 ± 14.2	12.7 ± 6.1	< 0.0001	18.4 ± 10.9	12.6 ± 6.7	< 0.0001	23.2 ± 14.0	14.9 ± 9.0	< 0.0001
Plasma Hcy µmol/L**	6.6 ± 2.7	7.6 ± 2.3	0.004	8.2 ± 3.6	8.5 ± 3.6	0.653	6.8 ± 2.7	8.4 ± 3.7	< 0.0001

* Reference value P-folate > 8 nmol/L (Architect, ABBOTT).

** Reference value P-Hcy < 15 µmol/L [17].

### Socioeconomic and lifestyle factors between infertile and fertile women

Principal component analyses, based on all women, showed that more highly educated women were physically more active and consumed more fruit and vegetables than women with lower levels of self-perceived health. The lower educated also had a higher mean BMI and more often were smokers (data not shown). When comparing infertile and fertile women, it was found that the infertile women had a better employment status (p < 0.0001), exercised more (p = 0.011) and smoked less (p = 0.012) than the fertile women. It was also noted that the infertile women were more often obese than the fertile women (p = 0.020).

### Folate intake levels

The mean food folate intake according to the 24-hour recalls was far lower than the current recommendation, with means ranging from 140 to 165 ug/day (Table 3). Previously published data show levels of mean intake of 223 ± 75 and 247 ± 92 ug/day for 18–30 and 30–44-year-old women, respectively [18].

### Folate status according to plasma folate and plasma homocysteine levels

Among all women mean plasma folate and plasma homocysteine levels were within the reference values (Table 3). Only 14% (n = 68) of women had plasma folate values under the reference value (< 8 nmol/L) and 2% (n = 11) had elevated plasma homocysteine levels (> 15 µmol/L). These levels were not related to folic acid supplement use or fertility status (data not shown).

### Validation of the questionnaire

To validate the questionnaire, data from 24-hour recall interviews were compared with data from questionnaires in 75 cases. Cohen’s kappa was 0.37, which was considered to represent fair agreement between the questionnaire results and the 24-hour recall data regarding folic acid supplement use.

The questionnaire was also tested vs. folate status, where the folate supplement users according to the questionnaire had significantly higher folate status (p < 0.0001) among infertile as well as fertile women and a lower homocysteine levels (p = 0.004) among the infertile participants (Table 3).

## Discussion

Infertile women were shown to use folic acid supplements to a higher extent in this study. Among fertile as well as infertile women in this study, high educational and employment status were found to be key factors for high compliance of folic acid supplement intake. Use of folic acid supplements, according to our questionnaire, also pointed at a better folate status in both infertile and fertile women. In infertile women, the supplement use was also associated with a higher mean dietary folate intake.

Fertility complications [11,19] as well as a higher socio-economic status [13,14,19–21] have previously been shown to be associated with more active folic acid supplement intake, which was also verified in the current study. In the present study, the infertile women had significantly higher mean daily folic acid intake than fertile women. Folic acid supplement users have previously been shown to have a generally better diet with higher intake of other dietary components, vitamins and minerals compared with non-users [20], a finding that was confirmed in this study.

It has previously been observed that women who already have children use fewer dietary supplements, especially folic acid supplements [5,20]. In the present study, less than half the studied fertile women took folic acid supplements and most had folate intakes far below the recommended. The reason for this might be that the fertile women were not pregnant or not planning to become pregnant, and therefore the incentives to use folic acid supplementation were lacking. However, it has been shown that approximately half of all pregnancies are unplanned [22,23], and therefore the importance of a sufficient intake of folate from foods or folic acid supplementation should be considered.

The overall analyses in the present study showed that physical activity was related to healthy food intake and a high level of education. When comparing infertile and fertile women, we found that the infertile women were physically more active and smoked less. These women also had better employment status than the fertile women. Women undergoing fertility treatment are in many cases well-educated and aware of the possible factors influencing the outcome of fertility treatment. Surprisingly, however, obesity, which is known to be associated with socioeconomic and lifestyle factors as well as with infertility [24], was more common in the infertile group. However, in these women obesity might be seen as a possible cause of infertility rather than reduction of the chance of a pregnancy.

When investigating diet and dietary supplement intake, the reliability of reported supplement use is crucial. It has been suggested that self-administered questionnaires work well for assessment of regular dietary supplement use [25]. Twenty-four-hour recall interviews allow more specific description of the actual food intake, while a questionnaire provides long-term information about usual intake [26]. The questionnaire used in this study showed only fair agreement (questionnaire vs. interview data) as regards the use of folic acid supplements. Overestimation of folic acid supplement use could have occurred in the questionnaire data, something that has been reported previously [9]. However, the generalizability of this result is limited since the data was from only 75 women in a sub-analysis.

A strength of our study lies in the assessment of folate status as well as folate intake including folic acid intake, which was calculated from information on dietary supplement use in addition to and not only through information on the diet as such. In addition, 24-hour recall interview data were used for validation of the questionnaire. For this validation we used data from 75 women, which can be considered to be a respectable sample size. However, lack of information on the bioavailability of naturally occurring folates [27,28] makes data collection regarding folate intake and its relation to folate status challenging and can in some respects be considered as a limitation of the study. Additionally, the 24-hour recall interviews were performed only on weekdays, which could have affected the collected data.

## Conclusions

It can be concluded that infertile women had better compliance to national advice on folic acid supplement use and they consequently also had a better folate status. Educational level and employment status were the most significant factors related to folic acid supplement use. Additionally, the used questionnaire for ‘usual’ use of supplements was shown to have fair validity in regards to folic acid supplement ‘actual’ use on the 24-hour recall days. There was also a significantly better folate status among the questionnaire assessed users of supplements.
